# Broad T Cell Targeting of Structural Proteins After SARS-CoV-2 Infection: High Throughput Assessment of T Cell Reactivity Using an Automated Interferon Gamma Release Assay

**DOI:** 10.3389/fimmu.2021.688436

**Published:** 2021-05-20

**Authors:** Isabel Brand, Leonard Gilberg, Jan Bruger, Mercè Garí, Andreas Wieser, Tabea M. Eser, Jonathan Frese, Mohamed I. M. Ahmed, Raquel Rubio-Acero, Jessica M. Guggenbuehl Noller, Noemi Castelletti, Jana Diekmannshemke, Sophie Thiesbrummel, Duc Huynh, Simon Winter, Inge Kroidl, Christiane Fuchs, Michael Hoelscher, Julia Roider, Sebastian Kobold, Michael Pritsch, Christof Geldmacher

**Affiliations:** ^1^ Division of Infectious Diseases and Tropical Medicine, University Hospital, Ludwig-Maximilians-Universität (LMU) Munich, Munich, Germany; ^2^ Division of Clinical Pharmacology, Department of Medicine IV, University Hospital, LMU Munich, Munich, Germany; ^3^ Department of Infectious Diseases, University Hospital, LMU Munich, Munich, Germany; ^4^ Institute of Computational Biology, Helmholtz Zentrum München – German Research Center for Environmental Health (HMGU), Neuherberg, Germany; ^5^ German Center for Infection Research (DZIF), Partner Site Munich, Munich, Germany; ^6^ Faculty of Business Administration and Economics, Bielefeld University, Bielefeld, Germany; ^7^ Center for Mathematics, Technische Universität München, Garching, Germany; ^8^ Center for International Health (CIH), University Hospital, LMU Munich, Munich, Germany; ^9^ German Center for Translational Cancer Research (DKTK), Partner Site Munich, Munich, Germany; ^10^ Unit for Clinical Pharmacology (EKLiP), Helmholtz Zentrum München – German Research Center for Environmental Health (HMGU), Neuherberg, Germany

**Keywords:** SARS-CoV-2, COVID-19, T cell response, interferon gamma release assay (IGRA), high through put

## Abstract

**Background:**

Adaptive immune responses to structural proteins of the virion play a crucial role in protection against coronavirus disease 2019 (COVID-19). We therefore studied T cell responses against multiple SARS-CoV-2 structural proteins in a large cohort using a simple, fast, and high-throughput approach.

**Methods:**

An automated interferon gamma release assay (IGRA) for the Nucleocapsid (NC)-, Membrane (M)-, Spike-C-terminus (SCT)-, and N-terminus-protein (SNT)-specific T cell responses was performed using fresh whole blood from study subjects with convalescent, confirmed COVID-19 (n = 177, more than 200 days post infection), exposed household members (n = 145), and unexposed controls (n = 85). SARS-CoV-2-specific antibodies were assessed using Elecsys^®^ Anti-SARS-CoV-2 (Ro-N-Ig) and Anti-SARS-CoV-2-ELISA (IgG) (EI-S1-IgG).

**Results:**

156 of 177 (88%) previously PCR confirmed cases were still positive by Ro-N-Ig more than 200 days after infection. In T cells, most frequently the M-protein was targeted by 88% seropositive, PCR confirmed cases, followed by SCT (85%), NC (82%), and SNT (73%), whereas each of these antigens was recognized by less than 14% of non-exposed control subjects. Broad targeting of these structural virion proteins was characteristic of convalescent SARS-CoV-2 infection; 68% of all seropositive individuals targeted all four tested antigens. Indeed, anti-NC antibody titer correlated loosely, but significantly with the magnitude and breadth of the SARS-CoV-2-specific T cell response. Age, sex, and body mass index were comparable between the different groups.

**Conclusion:**

SARS-CoV-2 seropositivity correlates with broad T cell reactivity of the structural virus proteins at 200 days after infection and beyond. The SARS-CoV-2-IGRA can facilitate large scale determination of SARS-CoV-2-specific T cell responses with high accuracy against multiple targets.

## Introduction

The coronavirus disease 2019 (COVID-19) pandemic, caused by the novel severe acute respiratory syndrome coronavirus 2 (SARS-CoV-2), started in December 2019. More than one year later, SARS-CoV-2 is still a serious threat to global health and a significant cause of mortality, especially in the elderly. Vaccines, mostly targeting the Spike protein of SARS-CoV-2 have been developed and approved at an unprecedented pace in history based on evidence for high efficacy (1). Yet, at the same time, restricting the vaccine target to a single protein or parts thereof also poses a risk of failure to immunization due to variants arising from natural viral mutations within the single protein of interest. In fact, newly emerging viral variants, such as B1.335, P.1, or B1.617 carrying mutations in the Spike protein, which potentially enhances the infectiousness of the virus, currently raise concerns that existing vaccines could lose or diminish their efficacy against these strains (2, 3). It was recognized early on that SARS-CoV-2 mounts a specific antibody based response that can protect from reinfections (4). As fundamental immunology teaches that antibody responses cannot be generated without a (T) cellular helper response, unsurprisingly, specific T cell responses were found in convalescent patients (5). Along these lines, a growing body of evidence has also recognized the existence and importance of cellular responses to SARS-CoV-2 infection in the clearance and later protection from reinfections (6). By nature, such responses are less convenient to measure and unfortunately there are no high throughput methods available to quantify SARS-CoV-2 specific T cell responses in patients.

Adaptive SARS-CoV-2-specific T cell responses likely have the capacity to protect the host at least from severe courses of COVID-19 upon reinfection even with the aforementioned immune escape variants. Upon reinfection, T cell recognition should nonetheless attenuate COVID-19 in those infected individuals (7). A broad T cell recognition of virus structural proteins can contribute to immune control even of highly variable viruses, such as HIV (8, 9), which easily escapes immune pressure inflicted by individual epitope-specific T cell responses (10).

Besides their role in adaptive immunity, SARS-CoV-2-specific T cell responses may also have a diagnostic value, as it has been reported that antibody levels wane faster than T cells. For example, SARS-CoV-1-specific antibody responses were short-lived and dropped below the limit of detection within 2 to 3 years (11, 12). As for SARS-CoV-2, antigen-specific antibody responses are not even detectable in all individuals, particularly in those with milder forms of COVID-19 (13–15).

Here, we report on SARS-CoV-2 specific T cell and antibody responses in a large cohort of study subjects with convalescent, PCR confirmed COVID-19, which did not require hospitalization, and in their exposed household members, as well as in unexposed controls. Using an automated, easy-to-use whole blood interferon gamma release assay (IGRA), we demonstrate that most individuals with serological evidence of convalescent SARS-CoV-2 infection, T and B cell reactivity against multiple structural proteins can be detected in peripheral blood at 200 days after infection/exposure and beyond.

## Materials and Methods

### Study Design, Study Subjects, and Specimen Collection

To establish a solid data basis for this study, we included study subjects in whose household at least one person has had a PCR confirmed SARS-CoV-2 infection. In May and June 2020, all households of Munich with at least one registered positive PCR for SARS-CoV-2 to date (more than 6000 households) were contacted by the responsible official authorities (City of Munich Health Department) and were provided information about COVID-19 related studies as well as contact details of the study center at the Division of Infectious Diseases and Tropical Medicine, University Hospital, LMU Munich, where upon more than 1000 households declared their interest in participating. Chronological enrollment took place from September 29, 2020 until January 27, 2021 of 177 PCR-positive individuals starting with the earliest registered PCR-positives and 145 of their household members. Furthermore, we randomly selected 40 households from a previously described population-based cohort study (KoCo 19) as controls (12, 16, 17) without any seropositive members on baseline as well as during follow up. A total of 36 of those households comprising 85 eligible members agreed to participate and were recruited during January 6-27, 2021. To investigate serology, cellular immune response and transmission, the study subjects of both groups were asked to provide a venous blood sample. Enrollment as well as specimen collection took place during household visits or at a central testing facility depending on study subjects’ preferences.

### Personal Information

Personal data of the study subjects was collected as described previously (18–20). In short, the mobile data collection tool OpenDataKit (ODK) was used to capture data during study visits by field workers on Android smartphones. Consecutively, study subjects completed household as well as personal questionnaires using a web-based application. Non responders were reminded first by email, and in case of continued non-response with a telephone reminder. Telephone interviews were offered to those who felt unable to complete the questionnaires online.

### Serologic Testing Methods

We determined antibody reactivity in plasma derived from ethylenediaminetetraacetic acid (EDTA)-coated blood tubes using Elecsys^®^ Anti-SARS-CoV-2 (Roche, Mannheim, Germany) hereafter Ro-N-Ig and Anti-SARS-CoV-2-ELISA (IgG) (Euroimmun, Lübeck, Germany), hereafter EI-S1-IgG. Testing was conducted in accordance with the manufacturers` recommendations. An optimized cutoff of 0.422 (instead of 1.0) for Ro-N-Ig was used to determine seropositivity in our study subjects, as described previously (18). An optimized cutoff of 1.015 (instead of 1.100) for EI-S1-IgG was only used in supplemental **Figure S1** as an additional marker for seropositivity in one subgroup.

### SARS-CoV-2 Interferon Gamma Release Assay (IGRA)

0.5 ml of fresh heparinized whole blood was added to each “Euroimmun” stimulator tube coated with SARS-CoV-2 specific antigens (Nucleocapsid protein, Spike-C-Terminus, Spike-N-Terminus and Membrane protein) and to negative and positive control tubes according to manufacturer instructions (Euroimmun, Lübeck, Germany). Tubes were inverted six times. After 16 to 20 hours of incubation at 37°C, 5% CO_2_ the samples were centrifuged at 12000 rcf for 10 minutes. The plasma supernatant was then transferred into a cryotube and stored at –80°C until testing. Interferon gamma (IFNγ) was detected automatically in the supernatants by an enzyme-linked immuno-sorbent assay (ELISA, Euroimmun, Lübeck, Germany) using the Euroimmun Analyzer I according to the manufacturer’s instructions. Using a standard curve, the IFNγ concentration was calculated. Background subtraction was carried out. Negative calculated values after background subtraction were set to 0 mIU/ml. All, but 3 out of 55 (5%) from the group exposed seropositive, 3 out of 90 (3%) from the group exposed seronegative and 5 out of 85 (6%) from the unexposed controls were stimulated with 3 antigens (NC, M and SCT). Study subjects, who were not stimulated with all antigenic regions provided too little blood. A subset of 232 (57%) study subjects was also stimulated with the SNT. All antigens were pools of synthetic 15mer peptides with 11 amino acid overlap (JPT Peptide Technologies), were based on the SARS-CoV-2 WUHAN isolate and were used at a final concentration of 5µg per stimulation. The Spike-N-terminal (PM-WCPV-S-2: P0DTC2) pool consisted of 158 peptides and the Spike-C-terminus (PM-WCPV-S-2: P0DTC2) of 157 peptides. The Nucleocapsid protein (PM-WCPV-NCAP: P0DTC9) contained 102 and the Membrane protein (PM-WCPV-VME: P0DTC5) 53 peptides. The utilized sequences for the peptides were previously used and described by others (21).

### Data Analysis

Data analysis and graphics were performed using the statistical software R (R Development Core Team, 2021) and the ggplot package (Wickham, 2016), as well as GraphPad Prism version 8 (GraphPad Software Inc.). Concentrations of IFNγ (mIU/ml) were log2 transformed for visual representation. The receiver operating characteristics (ROC) curve was used to define an optimized cutoff of IFNγ of 40 mIU/ml (**Tables S1A**, **B**). Differences in the IFNγ concentrations between the response to the antigenic regions were tested for significance using the Wilcoxon signed rank test and for differences in the IFNγ between EI-S1-IgG seropositive and seronegative study subjects the unpaired Wilcoxon test was used. Resulting p-values were adjusted for multiple testing using the Bonferroni correction. Spearman’s correlation coefficient (rho) was used to assess the correlation between Ro-N-Ig and the number of antigens detected. The flowchart was designed using (diagrams.net).

## Results

### Description of Study Population

**Figure 1:**
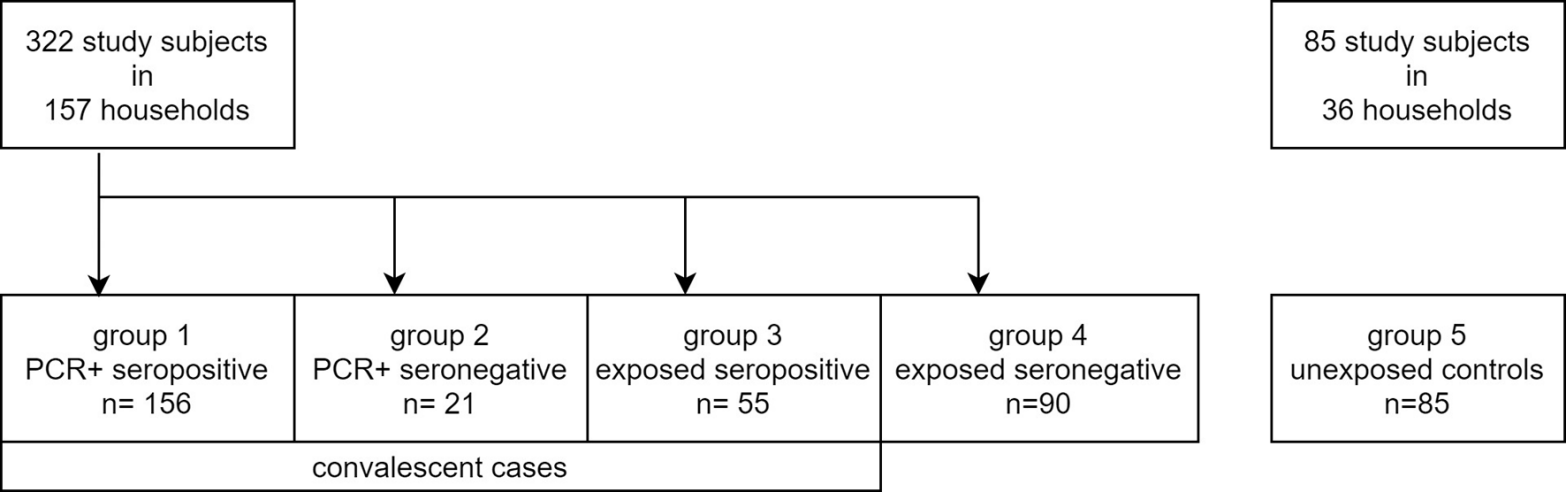
Flowchart of study subject groups.

A total of 182 households with 322 household members were recruited into this study (**Figure 1**). At least one resident of each household had been infected with SARS-CoV-2 and was diagnosed by a positive PCR result between March and April 2020 and registered by the City of Munich Health Department. These 322 study subjects were then tested using the IGRA, including individuals with PCR confirmed, convalescent SARS-CoV-2 infection. Only 11 of these study subjects had visited hospital outpatient facilities due to COVID-19 related symptoms, but none was hospitalized. All other COVID-19 cases in this study showed a mild course or did not report any symptoms at all.

In addition, 85 study subjects from 36 non-exposed households participating in the COVID-19 cohort Munich (KoCo19) were recruited as a control group and were also tested using the IGRA (**Figure 1**) (20, 22). These study subjects did not report contact to SARS-CoV-2 infected individuals and were previously tested twice for SARS-CoV-2 antibodies, being seronegative both times. At the time of blood collection, these individuals were tested again and remained seronegative. T cell responses against three SARS-CoV-2 structural antigens (Nucleocapsid (NC), Membrane protein (M), and Spike-C terminal region (SCT)) were tested for a total of 407 subjects (including the 85 controls) between October 2020 to January 2021 using a high throughput fresh whole blood IGRA. Within a subset of 232 study subjects (including 42 controls), reactivity to a fourth antigen (Spike-N terminal region (SNT)) was tested additionally. **Table 1** summarizes basic characteristics of these 407 individuals and shows that sex, age, and BMI were comparable between the groups with an overall median age of 41 years, a sex proportion of 51% females, and a median BMI of 23.9 kg/m². The median time between PCR testing and sample measurement was 243 (IQR 228.5 - 259.3) days in PCR-positive seropositive study subjects and 233 (IQR 223.0 - 244.5) days in PCR-positive seronegative ones.

**Table 1:** Overview on basic characteristics of the 407 study subjects.

	PCR-positive seropositive	PCR-positive seronegative		Exposed seropositive	Exposed seronegative	Unexposed controls	All study subjects
n	156	21		55	90	85	407
Sex							
Male	71 (45.5%)	15 (71.4%)		28 (50.9%)	43 (47.8%)	44 (51.8%)	201 (49.4%)
Female	85 (54.5%)	6 (28.6%)		27 (49.1%)	47 (52.2%)	41 (48.2%)	206 (50.6%)
Age (years)							
14-19	1(0.60%)	0 (0.00%)		6 (10.1%)	7 (7.80%)	11 (12.9%)	25 (6.10%)
20-34	32 (20.5%)	8 (38.1%)		20 (36.3%)	30 (33.3%)	15 (17.6%)	105 (25.8%)
35-49	68 (43.6%)	8 (38.1%)		13 (23.6%)	31 (34.4%)	36 (42.4%)	156 (38.3%)
50-64	44 (28.2%)	3 (14.3%)		11 (20.0%)	18 (20.0%)	16 (18.8%)	92 (22.6%)
65-79	11 (7.10%)	2 (9.50%)		5 (9.10%)	4 (4.40%)	3 (3.50%)	25 (6.10%)
80+	0 (0.00%)	0 (0.00%)		0 (0.00%)	0 (0.00%)	4 (4.70%)	4 (1.00%)
Median	44	39		35	40	43	41
Body Mass Index (kg/m²)							
< 18,5	2 (1.30%)	0 (0.00%)	2 (3.60%)		3 (3.30%)	2 (2.40%)	9 (2.20%)
18,5-25	89 (57.1%)	16 (76.2%)	32 (58.2%)		54 (60.0%)	47 (55.3%)	238 (58.5%)
25-30	54 (34.6%)	5 (23.8%)	11 (20.0%)		29 (32.2%)	24 (28.2%)	123 (30.2%)
> 30	11 (7.10%)	0 (0.00%)	9 (16.4%)		3 (3.30%)	11 (12.9%)	34 (8.40%)
NA	0 (0.00%)	0 (0.00%)	1 (1.80%)		1 (1.10%)	1 (1.20%)	3 (0.70%)
Median	24.2	24.2	23.7		23.3	24.1	23.9
Time from PCR to visit							
median (in days)	243	233					
IQR (in days)	228.5 - 259.3	223.0 - 244.5					

Sex, age and body mass index were comparable between the individual groups with an overall median age of 41 years, 51% females and BMI of 23.9 kg/m².

### T Cell Reactivity to Structural SARS-CoV-2 Proteins in PCR-Positive Convalescent Cases and Unexposed Controls

**Figure 2:**
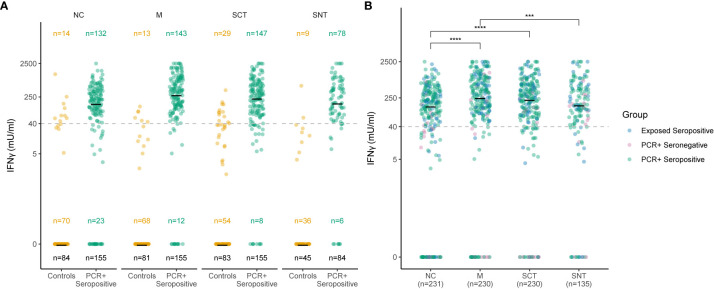
Sensitive and specific detection of T cell responses to four SARS-CoV-2 antigenic regions. Concentration of IFNγ in stimulated whole blood supernatants (y-axis) is shown as mIU/ml for the Nucleocapsid (NC), Membrane protein (M), Spike-C-Terminus (SCT) and Spike-N-Terminus (SNT). The numbers of subjects tested are indicated for each antigenic region and group. The black number at the bottom indicates overall number of study subjects in each group, numbers in the middle and the top show the number of subjects with IFNγ concentration of or above 0 mlU/ml, respectively. Cutoff of 40 mIU/ml IFNγ for T cell reactivity to an antigenic region is indicated as dashed line. Thick black lines mark median values. Each dot represents one study subject. Due to low blood volume, not all participants underwent the same analysis regarding the stimulation with the main three tested antigenic regions (NC, SCT and M). Therefore, sample sizes at each group between Antigens differ (see black sample size n below). 232 study subjects were also stimulated with SNT. **(A)** Ro-N-Ig seropositive subjects with PCR confirmed convalescent COVID-19 (green dots) were compared to negative controls from unexposed households (orange dots). **(B)** T cell recognition to the four tested structural antigens was compared for subjects with serological and/or PCR confirmed convalescent COVID-19. The p-values were calculated using Wilcoxon signed rank test. ^***^p ≤ 0.001, ^****^p ≤ 0.0001.

To define SARS-CoV-2-specific T cell reactivity with high sensitivity and specificity, we determined a single optimized cutoff for the concentration of IFNγ in stimulated whole blood supernatants for each of the tested SARS-CoV-2 antigenic regions NC, M, SCT, and SNT. To this end, we used PCR-positive seropositive cases as cases and unexposed individuals as controls (**Figure 2A**). ROC analysis confirmed an optimized cutoff at 40 mIU/ml IFNγ to define positive T cell responses against these antigenic regions. This resulted in a sensitivity of 82% and a specificity of 91% for T cell responses targeting the NC, a sensitivity of 88% and specificity of 94% for those targeting M protein, a sensitivity of 85% and specificity of 85% for those targeting SCT, and a sensitivity of 73% and specificity of 97% for those targeting SNT. As shown in **Figures 2A** and **S2**, for each of the antigenic regions most PCR-positive seropositive cases had IFNγ values of 40 mIU/ml or above upon *in vitro* stimulation with each of the antigens, whereas few non-exposed individuals had mounted such responses (10% to NC, 7% to M, 14% to SCT, 4% to SNT). SARS-CoV-2-specific T cells are most likely source of IFNγ production in this IGRA. We hence analyzed 10 convalescent SARS-CoV-2 patients (175 - 210 days post infection) using standard intracellular cytokine staining techniques after *in vitro* restimulation of fresh PBMCs with a Spike-specific or NC-specific peptide pool (**Figure S3** and **Table S2**). The source of antigen specific IFNγ production were mostly CD4 and sometimes CD8 T cells, which is consistent with previous reports (5, 23, 24). CD3 negative cells did not produce antigen specific IFNγ.

Next, we determined the concentration of IFNγ in stimulated supernatants for all study subjects with evidence of convalescent SARS-CoV-2 infection. All subjects were tested for NC, M and SCT antigens. 135 convalescent cases were stimulated with a fourth antigenic region, the SNT (**Figure 2B**). The median IFNγ concentration in stimulated supernatants for all study subjects with evidence of convalescent SARS-CoV-2 infection was 151 mIU/ml, 258 mIU/ml, 231 mIU/ml, and 162 mIU/ml for NC, M, SCT and SNT proteins, respectively (**Figure 2B**). The magnitude of the memory response towards the small M-protein was the strongest observed and significantly increased when compared to NC (p < 0.0001) and SNT (p < 0.001). There was also a significant increase in IFNγ production measured after stimulation with SCT when compared to NC (p < 0.0001). **Figure S2** shows IFNγ concentrations in mlU/ml against all four tested antigenic regions in all five groups (unexposed controls, exposed seronegatives, exposed seropositives, PCR-positive seronegatives and PCR-positives seropositives). We also highlight, that exposed seronegatives did not differ from unexposed controls with very narrow or non-existent SARS-CoV-2 T cell recognition. Overall, these results show that almost all individuals with evidence of convalescent SARS-CoV-2 infection mount memory T cell responses against structural proteins of the SARS-CoV-2 virion with the highest median IFNγ response magnitude determined for the small M protein.

### Broad T Cell Recognition of Structural SARS-CoV-2 Proteins at More Than 200 Days in Individuals With Convalescent SARS-CoV-2 Infection

**Figure 3:**
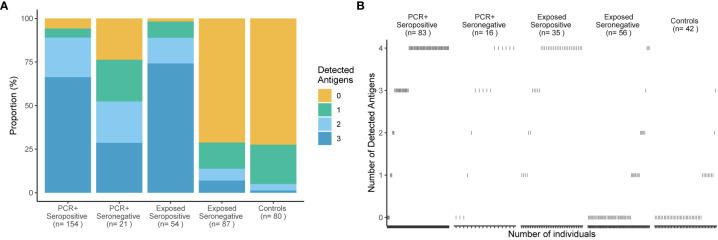
Broad T cell recognition of structural SARS-CoV-2 proteins in subjects with convalescent infection. Percentages of subjects (y-axis) who responded to 0, 1, 2 or 3 of 3 tested antigenic regions are shown in **(A)** for 5 groups delineated by PCR, Ro-N-Ig serostatus and SARS-CoV-2 exposure status. The number of study subjects in each group and the breadth of T cell recognition from 0 to 3 structural proteins are indicated for each group. A subgroup of 208 study subjects was tested with a fourth antigen - the Spike-N-Terminus **(B)**. The breadth of T cell recognition of the four tested antigens is shown for each of the 5 groups. Every dot represents an individual. Reactivity to Nucleocapsid Protein (NC), Spike-C-Terminus (SCT), and Membrane protein (M) was tested in stimulated whole blood supernatants.

**Figure 4:**
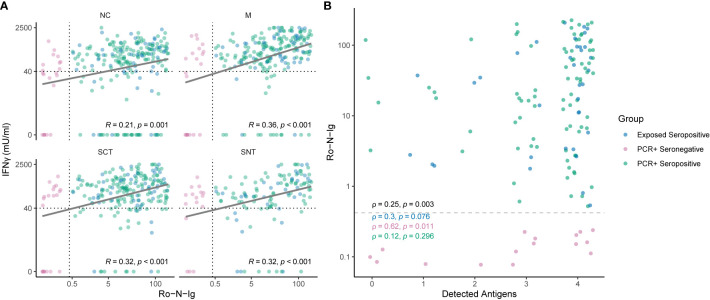
**(A)** Correlation of SARS-CoV-2-specific T cell reactivity to different antigenic regions and Roche-N-Ig titer. Shown are individuals of the convalescent group in different colors for each subgroup. Strong humoral immune response correlates with cellular reactivity to SARS-CoV-2 specific antigens measured in IFNγ (y-axis). Cutoffs for seropositivity and T cell reactivity to an antigenic region are indicated as dashed lines. Each dot represents one study subject. **(B)** Correlation of Roche-N-Ig and breadth reactivity of detected antigens. The plot shows Roche-N-Ig values for each individual recognizing 0, 1, 2, 3 or 4 antigenic regions. Only individuals which were tested for all four antigenic regions (Nucleocapsid NC, Spike-C-Terminus SCT, Spike-N-Terminus SNT and Membrane M) are shown. Cutoff for seropositivity is indicated by a dashed line. The p- value of non-zero correlation from all groups combined is shown in black. A low p-value means that the correlation is unlikely to be non-zero due to chance. Each dot represents one study subject.

The cutoff 40 mIU/ml was applied to define the breadth of SARS-CoV-2-specific T cell targeting of structural proteins in all groups stratified by serostatus, confirmatory PCR diagnoses and history of SARS-CoV-2 exposure. **Figure 3A** shows that, when tested against NC, M and SCT, most study subjects (70%) in the group PCR-positive seropositive targeted all tested SARS-CoV-2 specific antigenic regions and above 85% reacted to two of the three tested antigens. A similar pattern was detected for exposed seropositive study subjects, who were not confirmed by a positive PCR. A reduced breadth of SARS-CoV-2-specific T cell recognition was observed for the PCR-positive seronegative individuals. By contrast, above 70% of unexposed controls reacted to none of the tested antigens and the remaining ones typically reacted to only one of the tested SARS-CoV-2 antigenic regions. More than 70% of exposed seronegative study subjects also did not target any of the tested structural proteins, however the proportion of responders recognizing two or more antigens was increased, although not statistically significant (p = 0.055), when compared to unexposed controls. In individuals with four tested antigens (NC, M, SCT and SNT) a similar pattern was observed (**Figure 3B**); most subjects with evidence of convalescent SARS-CoV-2 infection reacted against all four tested antigens. Of note, we observed T cell reactivity to multiple antigenic regions in 75% (12 of 16 tested with four antigens) of study subjects who had been diagnosed by PCR but were seronegative at the time point of study inclusion. EI-S1-IgG against the SARS-CoV-2 S1 region was additionally measured in 17 PCR-positive, Ro-N-Ig-seronegative study subjects. 35% of these (6 of 17) had Spike-specific IgG antibody responses. Comparison of T cell reactivity to the M protein, but not the other 3 antigenic regions, differed significantly between these EI-S1-IgG-positive and EI-S1-IgG-negative study subjects (**Figure S1**). These findings suggest that many of these PCR-positive, seronegative study subjects are true convalescent COVID-19 cases and were not falsely diagnosed with COVID-19 in the past. However, we cannot exclude false positivity for a some of the subjects, who also did not have detectable Spike-specific IgG antibodies, nor a broader SARS-CoV-2 T cell response. Anti-NC antibody correlated loosely, but significantly with the magnitude and breadth of the SARS-CoV-2-specific T cell response (**Figure 4**). In summary, these results demonstrate broad T cell targeting of structural SARS-CoV-2 proteins long after convalescent infection in subjects with moderate, mild, or even asymptomatic SARS-CoV-2 infection. The high throughput interferon gamma release assay detected these responses with high sensitivity and specificity even in likely asymptomatic cases or in seronegative individuals.

## Discussion

Our study included study subjects of households, in which at least one member had a PCR confirmed SARS-CoV-2 infection between March and April 2020, including a subgroup of cases who had been infected, but had reported mild or no COVID-19-specific symptoms. Using a simple IGRA approach, we show that whole blood stimulation with different SARS-CoV-2 antigens can detect a broad cellular immune response to different structural proteins in convalescent individuals after moderate, mild, or completely asymptomatic COVID-19 at least 200 days after infection. In addition, the used approach provides high sensitivity and specificity. IFNγ production upon *in vitro* restimulation typically derives from CD4 and CD8 T cells and the tested structural antigens belong to the most immunodominant in acute SARS-CoV-2 infection (23). To prove this aspect, we used flow cytometry in 10 convalescent subjects more than 175 days after their reported infection, that also have been stimulated with structural SARS-CoV-2 proteins.

NC, M and S peptide pools were chosen for stimulation, because these represent structural proteins of the SARS-CoV-2 virion and were previously shown to induce high magnitude T cell responses (23). Because a previous study showed that T cell reactivity to the SNT peptide pool has high SARS-CoV-2 specificity (21), whereas the SCT peptide pool identified more non-SARS-CoV-2-specific T cell responses, the Spike protein was split into these two pools for the purpose of this study (23). Previous research has shown that CD4+ T cell responses are often stronger than corresponding CD8+ T cell responses, at least when using cryopreserved PBMC (5, 23, 24). Analyses of fresh, whole blood are the most direct way to assess antigen-specific cell function and avoid potential losses associated with PBMC cryopreservation. We therefore consider our approach to detect SARS-CoV-2 specific T cell responses as highly sensitive.

While most of exposed anti-nucleocapsid-seronegative study subjects did not mount T cell responses, there was a trend towards increased T cell recognition of multiple antigenic regions compared to the unexposed controls. This suggests that some formerly infected, now seronegative subjects retained SARS-CoV-2-specific T cell reactivity. Hence, assessment of a broad SARS-CoV-2-specific T cell response besides antibody responses increased detection of past SARS-CoV-2 transmission events in our study, which has been reported previously (16).

Another observation made was that most of the exposed seronegative study subjects did not differ from unexposed controls with very narrow or non-existent SARS-CoV-2 T cell recognition, probably because either no transmission event took place or narrow positive T cell responses to a single SARS-CoV-2 antigen could be the result of cross reactivity to other common cold coronaviruses (17, 21, 25). Indeed, 28% of unexposed controls had some narrow reactivity to SARS-CoV-2 structural proteins. Thereof, our results suggest that the current approach could be suited for identifying individuals with pre-existing cross-reactive T cell responses. This could facilitate studies on the potentially protective role of those T cells in SARS-CoV-2 infection.

Subjects with convalescent SARS-CoV-2 infection are well protected from reinfection, which correlates not only with anti-Spike antibodies, but also with anti-NC-antibodies (26). Our data show that anti-NC-seropositivity is also indicative of a broad T cell response against structural SARS-CoV-2 proteins in most seropositive individuals, including those individuals who did not report any COVID-19 specific symptoms. From the data it might also be concluded that in a certain fraction of subjects, specific T-cells are detected longer after the initial infection than antibodies tested with serological assays such as the one used here. A broad T cell recognition of virus structural proteins can contribute to immune control of variable viruses, such as HIV (8, 9). We therefore speculate that such broad virus-specific T cell immunity could contribute to reduce peak viral loads, to accelerate virus clearance and hence also reduce transmission risk and attenuate COVID-19 in case of reinfection with viral variants of concern, such as B1.335, P.1, or B1.617. Virus neutralization by antibodies is decreased for these variants (27), but to the best of our knowledge these have not escaped from T cell mediated immune pressure. Next generation polyvalent SARS-CoV-2 vaccines should therefore incorporate the comparatively small and immunogenic proteins M and NC to broaden vaccine-induced T cell recognition.

One limitation of this study is that we only included mild or asymptomatic COVID-19 convalescent cases and no severe ones. Another limitation is that the final antigen concentration differed between the different peptide pools on a per peptide level. We can therefore not exclude some effect of per peptide concentration on cellular responsiveness to the individual peptide pools. Nevertheless, this should not affect the overall results and interpretation of our study.

One strength of this study is that inclusion of SARS-CoV-2 exposed seropositive study subjects, who did not receive a PCR confirmed diagnosis, should have enriched for formerly infected subjects who had minimal or no COVID-19 specific symptoms and therefor did not get PCR tested. Unfortunately, due to a recall-bias, disease symptom reporting may have been incomplete after more than 200 days and hence we cannot conclude on differences in SARS-CoV-2-specific T cell memory between subjects with truly asymptomatic, very mild, or mild to moderate disease. It would be interesting to learn whether such individuals differ in their SARS-CoV-2-specific T cell memory and immunoreactivity in this assay. The major strength of this study relies on the combination of high throughput IGRA and automated serology platforms, that allowed us to be capable of investigating SARS-CoV-2-specific T and B cell responses for a large cohort in a limited amount of time. In addition, this also enabled us to analyze cellular responses to multiple structural virion proteins with high accuracy and in a diverse subset of individuals such as those with PCR or serologically confirmed convalescent COVID-19 as well as seronegative, exposed household members and unexposed controls. In conclusion, our results show that most subjects have broad T cell and B cell immunity at least 200 days after SARS-CoV-2 infection and beyond regardless of disease severity.

## KoCo19 Study Group Members

Emad Alamoudi, Jared Anderson, Abhishek Bakuli, Marc Becker, Franziska Bednarzki, Olimbek Bemirayev, Jessica Beyerl, Patrick Bitzer, Rebecca Boehnlein, Friedrich Caroli, Lorenzo Contento, Alina Czwienzek, Flora Deák, Maximilian N. Diefenbach, Gerhard Dobler, Jürgen Durner, Judith Eckstein, Philine Falk, Volker Fingerle, Felix Forster, Turid Frahnow, Guenter Froeschl, Otto Geisenberger, Kristina Gillig, Philipp Girl, Pablo Gutierrez, Anselm Haderer, Marlene Hannes, Jan Hasenauer, Tim Haselwarter, Alejandra Hernandes, Matthias Herrmann, Leah Hillari, Christian Hinske, Tim Hofberger, Sacha Horn, Kristina Huber, Christian Janke, Ursula Kappl, Antonia Kessler, Zohaib N. Khan, Johanna Kresin, Arne Kroidl, Magdalena Lang, Silvan Lange, Michael Laxy, Ronan Le Gleut, Reiner Leidl, Leopold Liedl, Xhovana Lucaj, Petra Mang, Alisa Markgraf, Rebecca Mayrhofer, Dafni Metaxa, Hannah Mueller, Katharina Mueller, Laura Olbrich, Ivana Paunovic, Claire Pleimelding, Michel Pletschette, Stephan Prueckner, Kerstin Puchinger, Peter Puetz, Katja Radon, Elba Raimundéz, Jakob Reich, Friedrich Riess, Camilla Rothe, Viktoria Ruci, Nicole Schaefer, Yannik Schaelte, Benedikt Schluse, Elmar Saathoff, Lara Schneider, Mirjam Schunk, Lars Schwettmann, Peter Sothmann, Kathrin Strobl, Jeni Tang, Fabian Theis, Verena Thiel, Jonathan von Lovenberg, Julia Waibel, Claudia Wallrauch, Roman Woelfel, Julia Wolff, Tobias Wuerfel, Sabine Zange, Eleftheria Zeggini, Anna Zielke

## Data Availability Statement

Data at individual level is not available due to protection of data privacy of our study subjects. However, data are accessible subject to data protection regulations upon reasonable request to the corresponding authors. Requests will be scientifically reviewed including the respective institutional review board if necessary and an appropriate data transfer agreement will have to be signed if approved. To facilitate reproducibility and reuse, the code used to perform the analyses and generate the figures has been made available on GitHub (https://github.com/koco19/IGRA).

## Ethics Statement

The study protocol was reviewed and approved by the Institutional Review Board of the Medical Faculty at Ludwig-Maximilians-University Munich, Germany under the project number 20-692 (vote of approval dated Sept. 21st, 2020) and 20-371 (vote of approval dated May 15th, 2020. Oral and written informed consent was obtained from all study subjects. For youths (ages 14 to 17) age-appropriate versions of the information and consent forms were used.

## Author Contributions

MH is the principal investigator and obtained most funds. MH, MP, CG, SK, and JR designed the study with help from JG and JF. SK and JR also obtained funds. Sample collection was led by JG, MP, JF, and IK. IB, LG, TE, MA, and DC performed the interferon gamma release assay. TE performed PBMC isolation and flow cytometry. JB generated study questionnaires. MG, JD, ST, JB, IB, LG, SW, NC, AW, and CF performed data cleaning and statistical analysis. LG, IB, and TE analyzed flow cytometry data. Data management and visualization was prepared by MG. High throughput serological testing was conducted by AW and RR-A. IB, LG, MP, JB, JF, JR, SK, MG, CF, and CG wrote the manuscript. All authors contributed to the article and approved the submitted version.

## Funding

This study was supported by the Program for Advancement of Corona Research Projects by the Bavarian Ministry for Science and Arts. The Koco19-Immu Study is funded by Bavarian State Ministry of Science and the Arts, University Hospital, LMU Munich, Helmholtz Centre Munich, University of Bonn, University of Bielefeld, German Ministry for Education and Research (prom. nr.: 01KI20271.) SK was supported by the Marie-Sklodowska-Curie Program Training Network for the Immunotherapy of Cancer and for Optimizing adoptive T cell therapy of cancer funded by the H2020 Program of the European Union (Grant 641549, to SK and grant 955575 to SK), the Hector foundation, the International Doctoral Program i Target: Immunotargeting of Cancer funded by the Elite Network of Bavaria (SK), the German Cancer Aid (SK), the Ernst-Jung-Stiftung (SK), LMU Munich’s Institutional Strategy LMUexcellent within the framework of the German Excellence Initiative (SK), the Bundesministerium für Bildung und Forschung Project Oncoattract and CONTRACT (SK), by the European Research Council Grant 756017, ARMOR-T (to SK), by the German Research Foundation (DFG to SK), by the Fritz-Bender-Foundation (to SK), by the Bavarian Ministry of Economic affairs (m4 award to SK), and the José-Carreras Foundation (to SK). This project has received funding from the European Union’s Horizon 2020 research and innovation programme under grant agreement No 101016167, ORCHESTRA (Connecting European Cohorts to Increase Common and Effective Response to SARS-CoV-2 Pandemic.

## Conflict of Interest

MP reports grant and non-financial support from Bavarian Ministry for Science and the Arts, non-financial support from Euroimmun, and non-financial support from Roche, during the conduct of the study. JB reports grant and non-financial support from Bavarian Ministry for Science and the Arts. IB and LG report non-financial support from Euroimmun. AW reports personal fees and non-financial support from Roche Diagnostics, non-financial support from Euroimmun, non-financial support from Viramed, non-financial support from Mikrogen, grants, non-financial support and other from German Center for Infection Research DZIF, grants and non-financial support from Government of Bavaria, non-financial support from BMW, non-financial support from Munich Police, from LGL, non-financial support and other from Accenture, during the conduct of the study; non-financial support and other from Bielefeld University, non-financial support and other from Bonn University, non-financial support and other from Helmholtz München, non-financial support and other from Bundeswehr (German army), personal fees and non-financial support from Dr.Box-Betrobox, non-financial support from Dr. Becker MVZ, outside the submitted work; In addition, AW has a patent Sample System for Diagnostics of SARS-CoV-2 pending to Wieser, Hoelscher, Becker.

The remaining authors declare that the research was conducted in the absence of any commercial or financial relationships that could be construed as a potential conflict of interest.

## Supplementary Material

Supplementary Table 1A Cutoff determination using ROC-Analysis. Shown are Sensitivity and Specificity for different amounts of interferon gamma for Nucleocapsid (NC) and Spike-C-terminus (SCT). Determined cutoff is indicated as a line. To define a general cutoff for a positive T cell response, we compared sensitivity and specificity of NC, SCT, SNT and M. 40mlU/ml showed the best combination of sensitivity and specificity when defining one single cutoff for all four antigenic regions.

Supplementary Table 1B Cutoff determination using ROC-Analysis. Shown are Sensitivity and Specificity for different amounts of interferon gamma for Spike-N-Terminus (SNT) and Membrane protein (M). Determined cutoff is indicated as a line. To define a general cutoff for a positive T cell response, we compared sensitivity and specificity of NC, SCT, SNT and M. 40mlU/ml showed the best combination of sensitivity and specificity when defining one single cutoff for all four antigenic regions.

Supplementary Table 2 Phenotypic characterization of IFNγ-positive cells responding to Spike- and Nucleocapsid peptide pools using intracellular cytokine staining. Shown are the percentages of parent cell populations for 10 study subjects. Columns from left to right according to the gating strategy. In total, 10 study subjects were tested after overnight *in vitro* restimulation of fresh PBMC at 180 days after SARS-CoV-2 symptom onset. A summary of these results and materials and methods are provided in **Supplementary Figure 3**.

Supplementary Figure 1 Comparison of IFNγ in PCR-positive, EI-S1-IgG seropositive, but Roche-N-Ig seronegative study subjects. Shown are individuals of the PCR-positive Ro-N-Ig seronegative group. The figure shows the concentration of IFNγ in stimulated whole blood supernatants (y-axis) in mlU/ml in 17 PCR-positive, EI-S1-IgG seropositive, but Ro-N-Ig seronegative study subjects. Six out of 17 (35%) Ro-N-Ig negative study subjects are EI-S1-IgG positive, arguing for being serologic Nucleocapsid non-responders. Comparison of T cell reactivity to the M protein, but not the other 3 antigenic regions, differed significantly between these EI-S1-IgG-positive and EI-S1-IgG-negative study subjects. Cutoff for EI-S1-IgG is indicated by a dashed line. Each dot represents one study subject. Wilcox Test p-values were calculated. In four study subjects EI-S1-IgG was not measured and only 12 out of 17 were tested with SNT. *p ≤ 0.05.

Supplementary Figure 2 Comparison of reactivity to the four structural antigenic regions in all study subjects delineated by exposure, PCR result and serostatus. Concentration of IFNγ in stimulated whole blood supernatants (y-axis) is shown as mIU/ml for the Nucleocapsid (NC), Membrane protein (M), Spike-C-Terminus (SCT) and Spike-N-Terminus (SNT). The numbers of subjects tested are indicated for each antigenic region and group. The black number at the bottom indicates overall number of study subjects in each group, numbers in the middle and the top show the number of subjects with IFNγ concentration of or above 0 mlU/ml, respectively. Cutoff of 40 mIU/ml IFNγ for T cell reactivity to an antigenic region is indicated as dashed line. Thick black lines mark median values. Each dot represents one study subject. Due to low blood volume, not all participants underwent the same analysis regarding the stimulation with the main three tested antigenic regions (NC, SCT and M). Therefore, sample sizes at each group between Antigens differ (see black sample size n below). 232 study subjects were also stimulated with SNT.

Supplementary Figure 3 Phenotypic characterization of IFNg+ cells responding to Spike- and Nucleocapsid peptide pools using intracellular cytokine staining. Shown are results for representative 5 study subjects (different columns). **(A)** shows CD3 versus IFNγ staining for cells of the lymphocyte gate. CD3+ T cells were then further delineated into CD4 **(B)** and CD8 **(C)** T cells. The stimulation antigen is indicated on the left. In total, 10 study subjects were tested 180 days after SARS-CoV-2 symptom onset. The associated data is provided in **Table S2**. In summary, out of the 10 subjects, 60% showed IFNγ+CD4+ T cells and 10% showed IFNγ+ CD8+ T cells upon *in vitro* restimulation with the Spike peptide pool. 90% and 50% had detectable IFNγ+ CD4+ and CD8 T cell responses to the Nucleocapsid peptide pool, respectively. 60% had IFNγ+CD4+ T cell reactivity to the Spike and Nucleocapsid peptide pools. A positive response is defined by a minimum of 15 cells/gate, a minimum of 0.01% IFNγ+ cells of CD4+ cells or CD8+ cells and a minimum of the double percentage of the negative control. The source of interferon gamma was determined by standard ICS procedures as follows; for the PBMC isolation, CPDA blood was centrifuged at 1285g for 10 minutes. After adding PBS, the suspension was filled into Leucosept tubes (Greiner) with Ficoll-Paque and centrifuged at 800g. PBMCs were harvested and directly prepared for flow cytometric analysis. PBMCs were incubated in complete medium at 37°C in 5% CO2 for 16h in the presence of either Nucleocapsid (NC), Spike-peptide pools (S), staphylococcus enterotoxin B (SEB) as positive control or nothing as negative control, and with a stimulation master mix containing costimulatory antibodies CD28, CD49d (Becton Dickinson, clones L293 and L25, respectively) and Brefeldin A (SIGMA). The PBMCs were then washed and stained with a surface antibody mix containing CD4-ECD (Beckman Coulter, clone SFCI12T4D1) and CD8–APC A750 (Beckman Coulter, clone B9.11) and incubated for 20 minutes in the dark. As the assays were performed on freshly isolated PBMC, no life/dead stain was added. After another washing step, the cells were permeabilized using pre-diluted FoxP3 Perm fixation buffer (ebioscience) and incubated of 25 minutes before adding diluted permeabilization buffer (ebioscience). PBMCs were then stained intracellularly using CD3–APC A700 (Beckman Coulter clone, UCHT1) and IFNγ-FITC (Biolegend, clone B27) for 30 minutes. Permeabilization buffer was added, and cells were acquired using a Cytoflex flow cytometry instrument (Beckman Coulter). Flow cytometry data analyses performed using FlowJo 10.7.2.

## References

[1] Polack FP Thomas SJ Kitchin N Absalon J Gurtman A Lockhart S. Safety and Efficacy of the BNT162b2 mRNA Covid-19 Vaccine. N Engl J Med (2020) 383(27):2603–15. 10.1056/NEJMoa2034577 PMC7745181 33301246

[2] Wang Z Schmidt F Weisblum Y Muecksch F Barnes CO Finkin S. mRNA Vaccine-Elicited Antibodies to SARS-CoV-2 and Circulating Variants. Nature (2021) 592:616–22. 10.3410/f.739524179.793585051 PMC8503938 33567448

[3] Wibmer CK Ayres F Hermanus T Madzivhandila M Kgagudi P Oosthuysen B. SARS-CoV-2 501Y.V2 Escapes Neutralization by South African COVID-19 Donor Plasma. Nat Med (2021) 27:622–5. 10.1101/2021.01.18.427166 33654292

[4] Zohar T Alter G. Dissecting Antibody-Mediated Protection Against SARS-Cov-2. Nat Rev Immunol (2020) 20(7):392–4. 10.1038/s41577-020-0359-5 PMC7278217 32514035

[5] Sekine T Perez-Potti A Rivera-Ballesteros O Stralin K Gorin JB Olsson A. Robust T Cell Immunity in Convalescent Individuals With Asymptomatic or Mild COVID-19. Cell (2020) 183(1):158–68 e14. 10.1101/2020.06.29.174888 32979941 PMC7427556

[6] Stephenson KE Le Gars M Sadoff J de Groot AM Heerwegh D Truyers C. Immunogenicity of the Ad26.COV2.S Vaccine for COVID-19. JAMA (2021) 325(15):1535–44. 10.1001/jama.2021.3645 PMC7953339 33704352

[7] Tarke A Sidney J Methot N Zhang Y Dan J Goodwin B. Negligible Impact of SARS-CoV-2 Variants on CD4+ and CD8+ T Cell Reactivity in COVID-19 Exposed Donors and Vaccinees. (2021). 10.1101/20210227433180 PMC8249675 34230917

[8] Geldmacher C Currier JR Herrmann E Haule A Kuta E McCutchan F. CD8 T-cell Recognition of Multiple Epitopes Within Specific Gag Regions is Associated With Maintenance of a Low Steady-State Viremia in Human Immunodeficiency Virus Type 1-Seropositive Patients. J Virol (2007) 81(5):2440–8. 10.1128/JVI.01847-06 PMC1865944 17182686

[9] Kiepiela P Ngumbela K Thobakgale C Ramduth D Honeyborne I Moodley E. CD8+ T-cell Responses to Different HIV Proteins Have Discordant Associations With Viral Load. Nat Med (2007) 13(1):46–53. 10.1038/nm1520 17173051

[10] Leslie AJ Pfafferott KJ Chetty P Draenert R Addo MM Feeney M. HIV Evolution: CTL Escape Mutation and Reversion After Transmission. Nat Med (2004) 10(3):282–9. 10.1038/nm992 14770175

[11] Channappanavar R Fett C Zhao J Meyerholz DK Perlman S. Virus-Specific Memory CD8 T Cells Provide Substantial Protection From Lethal Severe Acute Respiratory Syndrome Coronavirus Infection. J Virol (2014) 88(19):11034–44. 10.1128/JVI.01505-14 PMC4178831 25056892

[12] Tang F Quan Y Xin ZT Wrammert J Ma MJ Lv H. Lack of Peripheral Memory B Cell Responses in Recovered Patients With Severe Acute Respiratory Syndrome: A Six-Year Follow-Up Study. J Immunol (2011) 186(12):7264–8. 10.4049/jimmunol.0903490 21576510

[13] Gaebler C Wang Z Lorenzi JCC Muecksch F Finkin S Tokuyama M. Evolution of Antibody Immunity to SARS-Cov-2. Nature (2021) 591:639–44. 10.1038/s41586-021-03207-w 10.1038/s41586-021-03207-w PMC8221082 33461210

[14] Long QX Tang XJ Shi QL Li Q Deng HJ Yuan J. Clinical and Immunological Assessment of Asymptomatic SARS-CoV-2 Infections. Nat Med (2020) 26(8):1200–4. 10.1038/s41591-020-0965-6 32555424

[15] Mallapaty S. Will Antibody Tests for the Coronavirus Really Change Everything? Nature (2020) 580:571–2. 10.1038/d41586-020-01115-z 32313159

[16] Gallais F Velay A Nazon C Wendling MJ Partisani M Sibilia J. Intrafamilial Exposure to SARS-CoV-2 Associated With Cellular Immune Response Without Seroconversion, France. Emerg Infect Dis (2021) 27(1):113–21. 10.3201/eid2701.203611 PMC7774579 33261718

[17] Le Bert N Tan AT Kunasegaran K Tham CYL Hafezi M Chia A. SARS-CoV-2-Specific T Cell Immunity in Cases of COVID-19 and SARS, and Uninfected Controls. Nature (2020) 584(7821):457–62. 10.1038/s41586-020-2550-z 32668444

[18] Olbrich L Castelletti N Schälte Y Garí M Pütz P Bakuli A. A Serology Strategy for Epidemiological Studies Based on the Comparison of the Performance of Seven Different Test Systems - The Representative COVID-19 Cohort Munich. (2021). 10.1101/2021011321249735

[19] Pritsch M Radon K Bakuli A Le Gleut R Olbrich L Guggenbüehl Noller JM. Prevalence and Risk Factors of Infection in the Representative COVID-19 Cohort Munich. Inter J Environment Res Public Health (2021) 18(7):3572. 10.3390/ijerph18073572 PMC8038115 33808249

[20] Radon K Saathoff E Pritsch M Guggenbuhl Noller JM Kroidl I Olbrich L. Protocol of a Population-Based Prospective COVID-19 Cohort Study Munich, Germany (KoCo19). BMC Public Health (2020) 20(1):1036. 10.1186/s12889-020-09164-9 32605549 PMC7324773

[21] Braun J Loyal L Frentsch M Wendisch D Georg P Kurth F. SARS-CoV-2-Reactive T Cells in Healthy Donors and Patients With COVID-19. Nature (2020) 587(7833):270–4. 10.1038/s41586-020-2598-9 32726801

[22] KoCo19 (2021). Available at: http://www.klinikum.uni-muenchen.de/Abteilung-fuer-Infektions-und-Tropenmedizin/de/COVID-19/KoCo19/index.html.

[23] Grifoni A Weiskopf D Ramirez SI Mateus J Dan JM Moderbacher CR. Targets of T Cell Responses to SARS-CoV-2 Coronavirus in Humans With COVID-19 Disease and Unexposed Individuals. Cell (2020) 181(7):1489–501 e15. 10.1016/j.cell.2020.05.015 32473127 PMC7237901

[24] Sette A Crotty S. Adaptive Immunity to SARS-CoV-2 and COVID-19. Cell (2021) 184(4):861–80. 10.1016/j.cell.2021.01.007 PMC7803150 33497610

[25] Mateus J Grifoni A Tarke A Sidney J Ramirez SI Dan JM. Selective and Cross-Reactive SARS-CoV-2 T Cell Epitopes in Unexposed Humans. Science (2020) 370(6512):89–94. 10.1126/science.abd3871 32753554 PMC7574914

[26] Lumley SF O’Donnell D Stoesser NE Matthews PC Howarth A Hatch SB. Antibody Status and Incidence of SARS-CoV-2 Infection in Health Care Workers. N Engl J Med (2021) 384(6):533–40. 10.1056/NEJMoa2034545 PMC7781098 33369366

[27] Li Q Wu J Nie J Zhang L Hao H Liu S. The Impact of Mutations in SARS-CoV-2 Spike on Viral Infectivity and Antigenicity. Cell (2020) 182(5):1284–94 e9. 10.1016/j.cell.2020.07.012 32730807 PMC7366990

