# Supplementary material for: Broad T Cell Targeting of Structural Proteins After SARS-CoV-2 Infection: High Throughput Assessment of T Cell Reactivity Using an Automated Interferon Gamma Release Assay
Source: Front Immunol. 2021 May 20;12:688436. doi: 10.3389/fimmu.2021.688436 (PMC8173205; doi:10.3389/fimmu.2021.688436)
Supplement: Supplementary Table 1A — Cutoff determination using ROC-Analysis. Shown are Sensitivity and Specificity for different amounts of interferon gamma for Nucleocapsid (NC) and Spike-C-terminus (SCT). Determined cutoff is indicated as a line. To define a general cutoff for a positive T cell response, we compared sensitivity and specificity of NC, SCT, SNT and M. 40mlU/ml showed the best combination of sensitivity and specificity when defining one single cutoff for all four antigenic regions. [file Table_1.docx]

| **NC** |  |  | **SCT** |  |  |
| --- | --- | --- | --- | --- | --- |
|  | **Sensitivity (%)** | **Specificity (%)** |  | **Sensitivity (%)** | **Specificity (%)** |
| > 2.410 | 89.13 | 83.08 | > 1.190 | 95.65 | 63.08 |
| > 5.070 | 88.04 | 83.08 | > 2.635 | 95.65 | 64.62 |
| > 5.915 | 88.04 | 84.62 | > 3.040 | 95.65 | 66.15 |
| > 10.70 | 86.96 | 84.62 | > 4.895 | 95.65 | 67.69 |
| > 16.14 | 85.87 | 84.62 | > 7.365 | 94.57 | 67.69 |
| > 18.47 | 84.78 | 84.62 | > 8.415 | 94.57 | 69.23 |
| > 21.59 | 83.7 | 84.62 | > 9.240 | 94.57 | 70.77 |
| > 25.50 | 82.61 | 84.62 | > 10.00 | 93.48 | 70.77 |
| > 28.69 | 82.61 | 86.15 | > 11.12 | 93.48 | 72.31 |
| > 32.22 | 82.61 | 87.69 | > 13.79 | 92.39 | 72.31 |
| > 35.32 | 82.61 | 89.23 | > 16.95 | 92.39 | 73.85 |
| > 37.37 | 81.52 | 89.23 | > 18.71 | 92.39 | 75.38 |
| > 40.58 | 81.52 | 90.77 | > 19.32 | 91.3 | 75.38 |
| > 45.13 | 80.43 | 90.77 | > 24.78 | 90.22 | 75.38 |
| > 48.11 | 79.35 | 90.77 | > 30.47 | 90.22 | 76.92 |
| > 52.80 | 78.26 | 90.77 | > 31.61 | 89.13 | 76.92 |
| > 57.24 | 77.17 | 90.77 | > 34.25 | 89.13 | 78.46 |
| > 57.79 | 77.17 | 92.31 | > 36.21 | 86.96 | 78.46 |
| > 58.59 | 77.17 | 93.85 | > 36.26 | 86.96 | 80 |
| > 63.92 | 76.09 | 93.85 | > 36.84 | 86.96 | 83.08 |
| > 68.93 | 75 | 93.85 | > 37.71 | 86.96 | 84.62 |
| > 70.61 | 75 | 95.38 | > 39.19 | 85.87 | 84.62 |
| > 74.43 | 73.91 | 95.38 | > 40.38 | 84.78 | 84.62 |
| > 78.57 | 72.83 | 95.38 | > 42.26 | 84.78 | 86.15 |
| > 80.83 | 70.65 | 95.38 | > 46.27 | 83.7 | 86.15 |
| > 85.73 | 69.57 | 95.38 | > 53.19 | 83.7 | 87.69 |
| > 90.61 | 68.48 | 95.38 | > 58.80 | 82.61 | 87.69 |
| > 92.04 | 67.39 | 95.38 | > 62.28 | 81.52 | 87.69 |
| > 98.30 | 66.3 | 95.38 | > 65.01 | 81.52 | 89.23 |
| > 103.6 | 65.22 | 95.38 | > 68.25 | 80.43 | 89.23 |
|  |  |  | > 71.60 | 79.35 | 89.23 |
|  |  |  | > 72.47 | 78.26 | 89.23 |
|  |  |  | > 73.55 | 77.17 | 89.23 |
|  |  |  | > 75.36 | 77.17 | 90.77 |
|  |  |  | > 77.41 | 76.09 | 90.77 |
|  |  |  | > 78.51 | 75 | 90.77 |
|  |  |  | > 81.58 | 73.91 | 90.77 |
|  |  |  | > 85.57 | 72.83 | 90.77 |
|  |  |  | > 87.62 | 71.74 | 90.77 |
|  |  |  | > 88.80 | 71.74 | 92.31 |
|  |  |  | > 92.89 | 71.74 | 93.85 |
|  |  |  | > 97.85 | 70.65 | 93.85 |
|  |  |  | > 102.4 | 69.57 | 93.85 |

**Table S1A. Cutoff determination using ROC-Analysis.** Shown are Sensitivity and Specificity for different amounts of interferon gamma for Nucleocapsid (NC) and Spike-C-terminus (SCT). Determined cutoff is indicated as a line. To define a general cutoff for a positive T cell response, we compared sensitivity and specificity of NC, SCT, SNT and M. 40mlU/ml showed the best combination of sensitivity and specificity when defining one single cutoff for all four antigenic regions.

| **SNT** |  |  | **M** |  |  |
| --- | --- | --- | --- | --- | --- |
|  | **Sensitivity (%)** | **Specificity (%)** |  | **Sensitivity (%)** | **Specificity (%)** |
| > 1.665 | 81.16 | 86.49 | > 2.555 | 93.48 | 84.62 |
| > 5.090 | 81.16 | 89.19 | > 6.810 | 92.39 | 86.15 |
| > 8.980 | 79.71 | 89.19 | > 8.855 | 92.39 | 87.69 |
| > 12.53 | 79.71 | 91.89 | > 10.55 | 91.3 | 87.69 |
| > 14.69 | 78.26 | 91.89 | > 12.62 | 91.3 | 89.23 |
| > 16.41 | 76.81 | 91.89 | > 15.00 | 91.3 | 90.77 |
| > 19.59 | 76.81 | 94.59 | > 18.80 | 91.3 | 92.31 |
| > 25.15 | 76.81 | 97.3 | > 22.81 | 90.22 | 92.31 |
| > 33.14 | 75.36 | 97.3 | > 26.23 | 89.13 | 92.31 |
| > 38.49 | 73.91 | 97.3 | > 30.19 | 88.04 | 92.31 |
| > 42.36 | 72.46 | 97.3 | > 39.84 | 88.04 | 93.85 |
| > 47.27 | 71.01 | 97.3 | > 47.45 | 86.96 | 93.85 |
| > 49.09 | 69.57 | 97.3 | > 48.36 | 85.87 | 93.85 |
| > 50.49 | 68.12 | 97.3 | > 50.80 | 85.87 | 95.38 |
| > 53.02 | 66.67 | 97.3 | > 55.87 | 84.78 | 95.38 |
| > 57.16 | 65.22 | 97.3 | > 59.20 | 83.7 | 95.38 |
| > 66.49 | 62.32 | 97.3 | > 60.86 | 83.7 | 96.92 |
| > 73.28 | 60.87 | 97.3 | > 64.15 | 82.61 | 96.92 |
| > 74.56 | 59.42 | 97.3 | > 72.46 | 81.52 | 96.92 |
| > 78.07 | 57.97 | 97.3 | > 80.17 | 80.43 | 96.92 |
| > 81.01 | 56.52 | 97.3 | > 82.61 | 79.35 | 96.92 |
| > 88.34 | 55.07 | 97.3 | > 88.37 | 78.26 | 96.92 |
| > 102.9 | 53.62 | 97.3 | > 92.99 | 78.26 | 98.46 |
|  |  |  | > 93.29 | 77.17 | 98.46 |
|  |  |  | > 103.4 | 76.09 | 98.46 |

**Table S1B. Cutoff determination using ROC-Analysis.** Shown are Sensitivity and Specificity for different amounts of interferon gamma for Spike-N-Terminus (SNT) and Membrane protein (M). Determined cutoff is indicated as a line. To define a general cutoff for a positive T cell response, we compared sensitivity and specificity of NC, SCT, SNT and M. 40mlU/ml showed the best combination of sensitivity and specificity when defining one single cutoff for all four antigenic regions.
