# Supplementary material for: Broad T Cell Targeting of Structural Proteins After SARS-CoV-2 Infection: High Throughput Assessment of T Cell Reactivity Using an Automated Interferon Gamma Release Assay
Source: Front Immunol. 2021 May 20;12:688436. doi: 10.3389/fimmu.2021.688436 (PMC8173205; doi:10.3389/fimmu.2021.688436)
Supplement: Supplementary Table 2 — Phenotypic characterization of IFNγ-positive cells responding to Spike- and Nucleocapsid peptide pools using intracellular cytokine staining. Shown are the percentages of parent cell populations for 10 study subjects. Columns from left to right according to the gating strategy. In total, 10 study subjects were tested after overnight in vitro restimulation of fresh PBMC at 180 days after SARS-CoV-2 symptom onset. A summary of these results and materials and methods are provided in Supplementary Figure 3 . [file Table_2.docx]

|  | **Lymphocytes** | **Singlets** | **CD3+** | **CD3+CD4+** | **CD4+IFNg+** | **CD3+CD8+** | **CD8+IFNg+** |
| --- | --- | --- | --- | --- | --- | --- | --- |
| **Study subject 1** |  |  |  |  |  |  |  |
| Nucleocapsid | 38.200% | 89.900% | 75.200% | 67.900% | 0.034% | 23.900% | 0.140% |
| Spike | 40.200% | 85.500% | 77.500% | 66.300% | 0.014% | 25.900% | 0.009% |
| positive control | 47.900% | 94.100% | 74.500% | 64.200% | 4.740% | 25.800% | 2.580% |
| negative control | 41.200% | 78.600% | 74.200% | 66.900% | 0.000% | 25.600% | 0.000% |
| **Study subject 2** |  |  |  |  |  |  |  |
| Nucleocapsid | 65.700% | 93.400% | 80.300% | 57.400% | 0.032% | 28.400% | 0.008% |
| Spike | 66.500% | 92.700% | 81.200% | 55.300% | 0.016% | 29.200% | 0.011% |
| positive control | 61.900% | 79.900% | 78.500% | 56.300% | 2.360% | 27.400% | 1.440% |
| negative control | 66.600% | 90.700% | 78.300% | 57.800% | 0.002% | 28.300% | 0.000% |
| **Study subject 3** |  |  |  |  |  |  |  |
| Nucleocapsid | 62.200% | 92.000% | 80.800% | 51.200% | 0.028% | 38.900% | 0.015% |
| Spike | 68.300% | 95.400% | 79.500% | 46.300% | 0.026% | 43.300% | 0.001% |
| positive control | 66.300% | 95.000% | 79.300% | 40.800% | 2.240% | 33.500% | 2.230% |
| negative control | 68.100% | 95.300% | 78.700% | 51.200% | 0.002% | 41.100% | 0.002% |
| **Study subject 4** |  |  |  |  |  |  |  |
| Nucleocapsid | 48.100% | 89.500% | 79.100% | 50.300% | 0.060% | 41.700% | 0.027% |
| Spike | 60.700% | 83.500% | 76.500% | 50.100% | 0.007% | 42.000% | 0.001% |
| positive control | 62.700% | 81.500% | 70.900% | 49.400% | 4.670% | 40.100% | 4.680% |
| negative control | 61.300% | 78.500% | 73.500% | 49.900% | 0.003% | 42.000% | 0.002% |
| **Study subject 5** |  |  |  |  |  |  |  |
| Nucleocapsid | 68.100% | 87.900% | 68.200% | 70.600% | 0.094% | 21.400% | 0.005% |
| Spike | 69.300% | 88.900% | 70.300% | 69.100% | 0.021% | 22.600% | 0.002% |
| positive control | 66.300% | 86.700% | 69.500% | 69.200% | 2.320% | 22.300% | 7.740% |
| negative control | 67.700% | 86.400% | 69.200% | 69.500% | 0.107% | 22.600% | 0.000% |
| **Study subject 6** |  |  |  |  |  |  |  |
| Nucleocapsid | 74.800% | 90.600% | 79.400% | 75.100% | 0.018% | 16.400% | 0.003% |
| Spike | 71.200% | 94.400% | 77.200% | 72.500% | 0.008% | 17.200% | 0.002% |
| positive control | 73.000% | 92.300% | 82.400% | 73.300% | 0.810% | 17.000% | 0.670% |
| negative control | 75.300% | 95.000% | 76.300% | 72.900% | 0.000% | 17.600% | 0.001% |
| **Study subject 7** |  |  |  |  |  |  |  |
| Nucleocapsid | 60.400% | 87.000% | 83.400% | 49.300% | 0.026% | 41.300% | 0.020% |
| Spike | 65.300% | 88.600% | 82.700% | 50.200% | 0.059% | 40.600% | 0.004% |
| positive control | 65.100% | 88.400% | 86.600% | 50.800% | 2.490% | 39.300% | 4.830% |
| negative control | 65.700% | 86.800% | 82.500% | 50.200% | 0.012% | 41.000% | 0.013% |
| **Study subject 8** |  |  |  |  |  |  |  |
| Nucleocapsid | 48.500% | 88.400% | 66.500% | 79.500% | 0.031% | 14.900% | 0.200% |
| Spike | 53.200% | 91.400% | 69.000% | 79.000% | 0.010% | 15.700% | 0.007% |
| positive control | 49.900% | 84.500% | 68.300% | 75.800% | 2.590% | 14.500% | 6.030% |
| negative control | 46.000% | 84.500% | 66.000% | 80.000% | 0.008% | 13.900% | 0.000% |
| **Study subject 9** |  |  |  |  |  |  |  |
| Nucleocapsid | 61.500% | 94.300% | 67.800% | 64.800% | 0.027% | 29.100% | 0.022% |
| Spike | 60.900% | 92.300% | 67.700% | 64.700% | 0.008% | 28.500% | 0.002% |
| positive control | 60.100% | 89.900% | 69.500% | 67.300% | 1.330% | 26.300% | 2.220% |
| negative control | 64.200% | 94.400% | 65.000% | 65.600% | 0.001% | 27.600% | 0.001% |
| **Study subject 10** |  |  |  |  |  |  |  |
| Nucleocapsid | 59.500% | 89.100% | 73.300% | 71.900% | 0.017% | 24.100% | 0.005% |
| Spike | 63.300% | 87.400% | 71.300% | 71.600% | 0.020% | 24.600% | 0.007% |
| positive control | 62.400% | 68.800% | 70.000% | 75.400% | 2.570% | 20.400% | 2.630% |
| negative control | 64.000% | 87.600% | 69.400% | 73.600% | 0.003% | 22.600% | 0.003% |

**Table S2. Phenotypic characterization of IFNγ-positive cells responding to Spike- and Nucleocapsid peptide pools using intracellular cytokine staining.** Shown are the percentages of parent cell populations for 10 study subjects. Columns from left to right according to the gating strategy. In total, 10 study subjects were tested after overnight in vitro restimulation of fresh PBMC at 180 days after SARS-CoV-2 symptom onset. A summary of these results and materials and methods are provided in supplementary figure 3.
