# Supplementary figures and images for: Broad T Cell Targeting of Structural Proteins After SARS-CoV-2 Infection: High Throughput Assessment of T Cell Reactivity Using an Automated Interferon Gamma Release Assay
Source: Front Immunol. 2021 May 20;12:688436. doi: 10.3389/fimmu.2021.688436 (PMC8173205; doi:10.3389/fimmu.2021.688436)

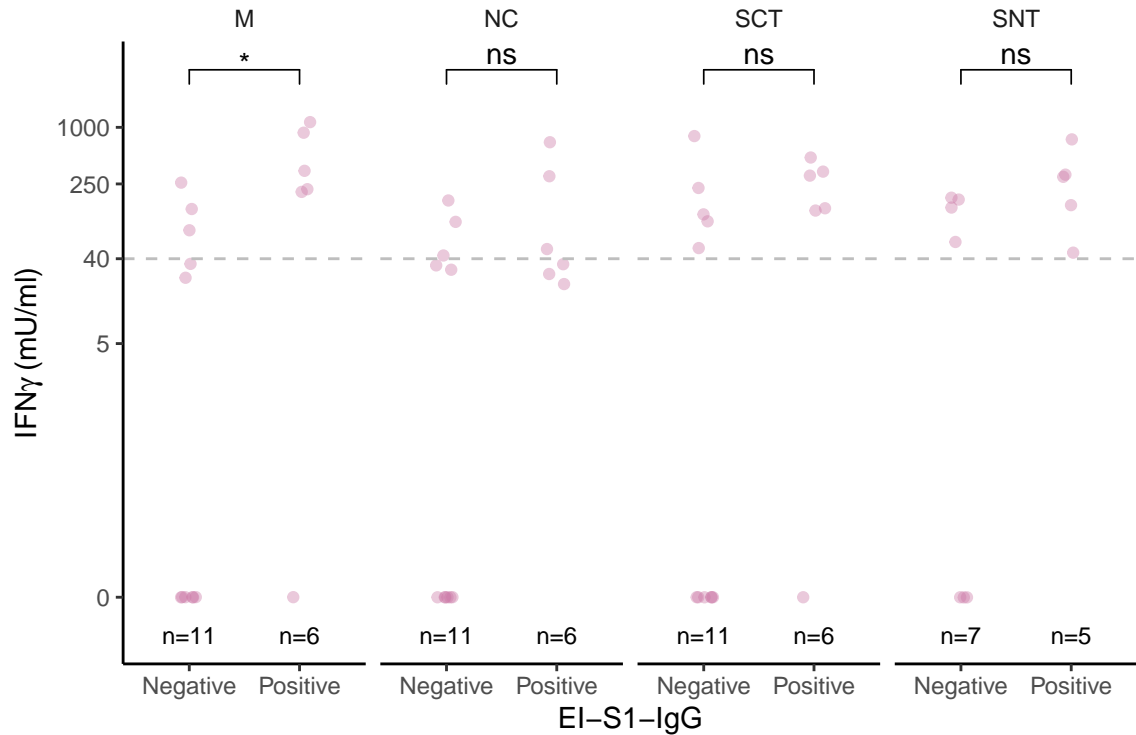

Supplement: Supplementary Figure 1 — Comparison of IFNγ in PCR-positive, EI-S1-IgG seropositive, but Roche-N-Ig seronegative study subjects. Shown are individuals of the PCR-positive Ro-N-Ig seronegative group. The figure shows the concentration of IFNγ in stimulated whole blood supernatants (y-axis) in mlU/ml in 17 PCR-positive, EI-S1-IgG seropositive, but Ro-N-Ig seronegative study subjects. Six out of 17 (35%) Ro-N-Ig negative study subjects are EI-S1-IgG positive, arguing for being serologic Nucleocapsid non-responders. Comparison of T cell reactivity to the M protein, but not the other 3 antigenic regions, differed significantly between these EI-S1-IgG-positive and EI-S1-IgG-negative study subjects. Cutoff for EI-S1-IgG is indicated by a dashed line. Each dot represents one study subject. Wilcox Test p-values were calculated. In four study subjects EI-S1-IgG was not measured and only 12 out of 17 were tested with SNT. *p ≤ 0.05. [file Image_1.pdf]

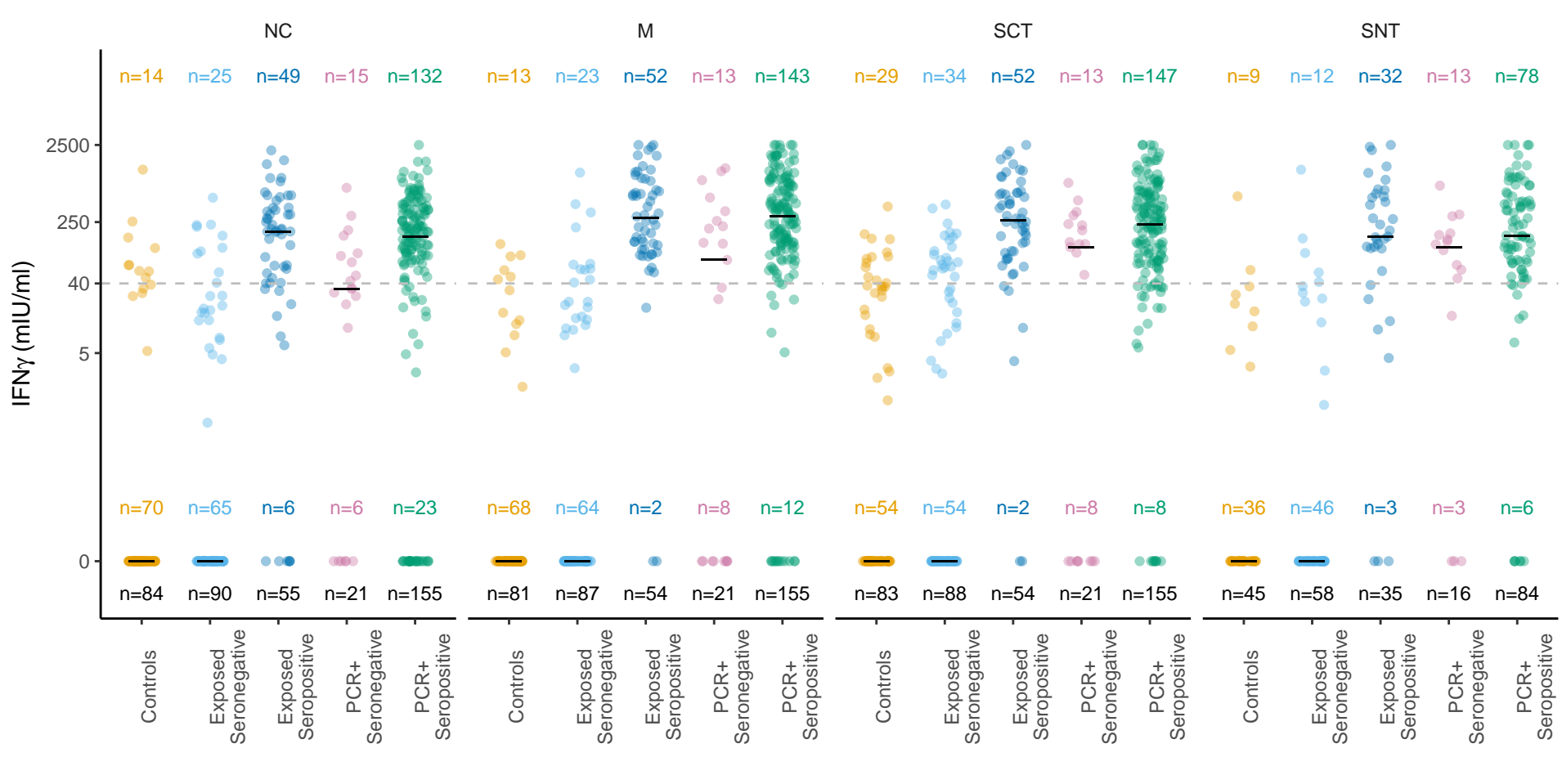

Supplement: Supplementary Figure 2 — Comparison of reactivity to the four structural antigenic regions in all study subjects delineated by exposure, PCR result and serostatus. Concentration of IFNγ in stimulated whole blood supernatants (y-axis) is shown as mIU/ml for the Nucleocapsid (NC), Membrane protein (M), Spike-C-Terminus (SCT) and Spike-N-Terminus (SNT). The numbers of subjects tested are indicated for each antigenic region and group. The black number at the bottom indicates overall number of study subjects in each group, numbers in the middle and the top show the number of subjects with IFNγ concentration of or above 0 mlU/ml, respectively. Cutoff of 40 mIU/ml IFNγ for T cell reactivity to an antigenic region is indicated as dashed line. Thick black lines mark median values. Each dot represents one study subject. Due to low blood volume, not all participants underwent the same analysis regarding the stimulation with the main three tested antigenic regions (NC, SCT and M). Therefore, sample sizes at each group between Antigens differ (see black sample size n below). 232 study subjects were also stimulated with SNT. [file Image_2.pdf]

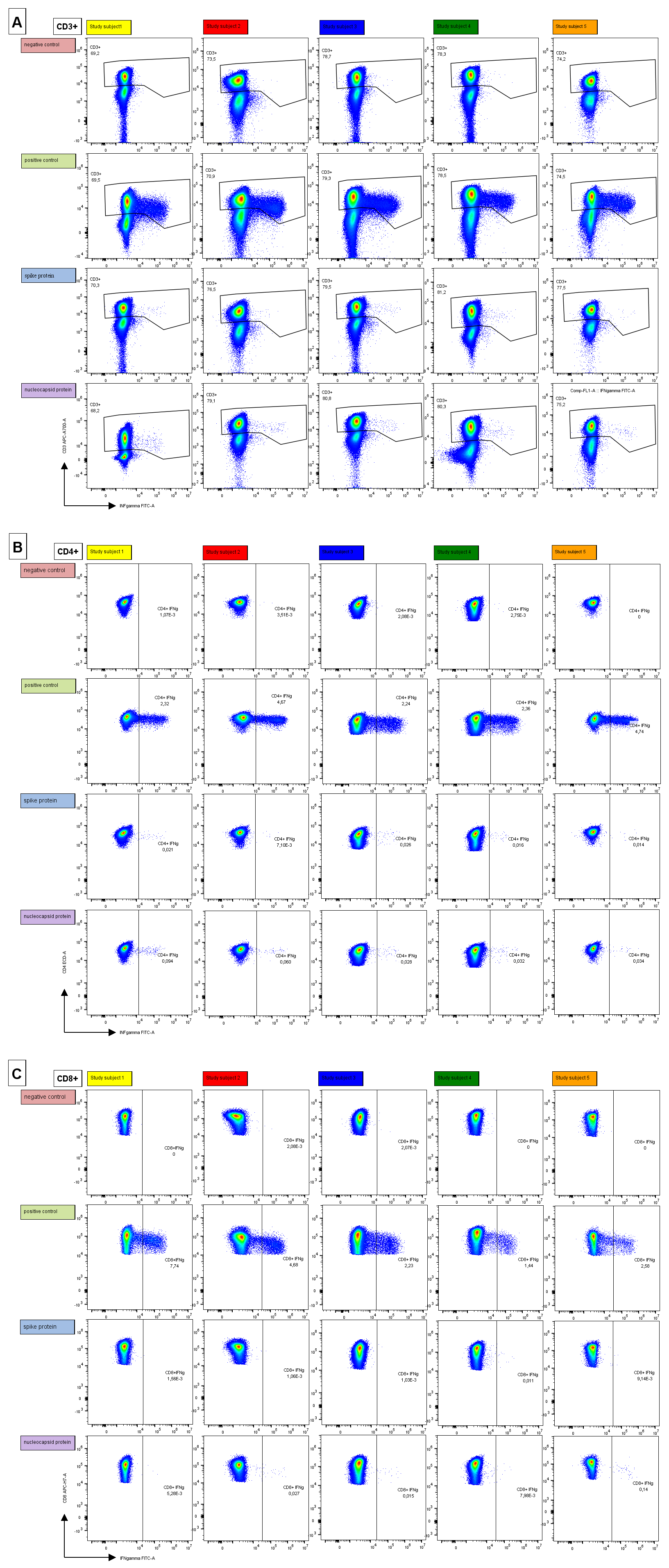

Supplement: Supplementary Figure 3 — Phenotypic characterization of IFNg+ cells responding to Spike- and Nucleocapsid peptide pools using intracellular cytokine staining. Shown are results for representative 5 study subjects (different columns). (A) shows CD3 versus IFNγ staining for cells of the lymphocyte gate. CD3+ T cells were then further delineated into CD4 (B) and CD8 (C) T cells. The stimulation antigen is indicated on the left. In total, 10 study subjects were tested 180 days after SARS-CoV-2 symptom onset. The associated data is provided in Table S2 . In summary, out of the 10 subjects, 60% showed IFNγ+CD4+ T cells and 10% showed IFNγ+ CD8+ T cells upon in vitro restimulation with the Spike peptide pool. 90% and 50% had detectable IFNγ+ CD4+ and CD8 T cell responses to the Nucleocapsid peptide pool, respectively. 60% had IFNγ+CD4+ T cell reactivity to the Spike and Nucleocapsid peptide pools. A positive response is defined by a minimum of 15 cells/gate, a minimum of 0.01% IFNγ+ cells of CD4+ cells or CD8+ cells and a minimum of the double percentage of the negative control. The source of interferon gamma was determined by standard ICS procedures as follows; for the PBMC isolation, CPDA blood was centrifuged at 1285g for 10 minutes. After adding PBS, the suspension was filled into Leucosept tubes (Greiner) with Ficoll-Paque and centrifuged at 800g. PBMCs were harvested and directly prepared for flow cytometric analysis. PBMCs were incubated in complete medium at 37°C in 5% CO2 for 16h in the presence of either Nucleocapsid (NC), Spike-peptide pools (S), staphylococcus enterotoxin B (SEB) as positive control or nothing as negative control, and with a stimulation master mix containing costimulatory antibodies CD28, CD49d (Becton Dickinson, clones L293 and L25, respectively) and Brefeldin A (SIGMA). The PBMCs were then washed and stained with a surface antibody mix containing CD4-ECD (Beckman Coulter, clone SFCI12T4D1) and CD8–APC A750 (Beckman Coulter, clone B9.11) and incubated for 20 minutes i [file Image_3.png]
